# CT imaging changes of corona virus disease 2019(COVID-19): a multi-center study in Southwest China

**DOI:** 10.1186/s12967-020-02324-w

**Published:** 2020-04-06

**Authors:** Xiaoming Li, Wenbing Zeng, Xiang Li, Haonan Chen, Linping Shi, Xinghui Li, Hongnian Xiang, Yang Cao, Hui Chen, Chen Liu, Jian Wang

**Affiliations:** 1 Department of Radiology, First Affiliated Hospital to Army Medical University, Chongqing, China; 2 Department of Radiology, Three Gorges Central Hospital, Chongqing, China; 3 Department of Radiology, Hubei Provincial Corps Hospital Chinese People’s Armed Police Forces, Wuhan, Hubei China; 4 Department of Radiology, Affiliated Hospital of North Sichuan Medical College, Nanchong, Sichuan China; 5 Department of Radiology, Chongqing Wuxi County People’s Hospital, Chongqing, China; 6 Department of Radiology, Dianjiang People’s Hospital of Chongqing, Chongqing, China

**Keywords:** Coronavirus, The chest, Computed tomography, Pneumonia, Evolvement

## Abstract

**Background:**

Since the first case of a coronavirus disease 2019 (COVID-19) infection pneumonia was detected in Wuhan, China, a series of confirmed cases of the COVID-19 were found in Southwest China. The aim of this study was to describe the imaging manifestations of hospitalized patients with confirmed COVID-19 infection in southwest China.

**Methods:**

In this retrospective study, data were collected from 131 patients with confirmed coronavirus disease 2019 (COVID-19) from 3 Chinese hospitals. Their common clinical manifestations, as well as characteristics and evolvement features of chest CT images, were analyzed.

**Results:**

A total of 100 (76%) patients had a history of close contact with people living in Wuhan, Hubei. The clinical manifestations of COVID-19 included cough, fever. Most of the lesions identified in chest CT images were multiple lesions of bilateral lungs, lesions were more localized in the peripheral lung, 109 (83%) patients had more than two lobes involved, 20 (15%) patients presented with patchy ground glass opacities, patchy ground glass opacities and consolidation of lesions co-existing in 61 (47%) cases. Complications such as pleural thickening, hydrothorax, pericardial effusion, and enlarged mediastinal lymph nodes were detected but only in rare cases. For the follow-up chest CT examinations (91 cases), We found 66 (73%) cases changed very quickly, with an average of 3.5 days, 25 cases (27%) presented absorbed lesions, progression was observed in 41 cases (46%), 25 (27%) cases showed no significant changes.

**Conclusion:**

Chest CT plays an important role in diagnosing COVID-19. The imaging pattern of multifocal peripheral ground glass or mixed consolidation is highly suspicious of COVID-19, that can quickly change over a short period of time.

## Background

An epidemic of the 2019 novel coronavirus (2019-nCoV) erupted in Wuhan, Hubei Province of China at the end of December 2019, and has quickly spread across the country, including overseas. As of February 23, 2020, 77,041 confirmed cases have been reported in China, with a cumulative death toll of 2445. As a newly identified member of the coronavirus-beta subfamily, the virus shares more than 85% homology with the bat severe acute respiratory syndrome (SARS)-like coronavirus (bat-SL-CoVZC45), belonging to the same class as the SARS-CoV found in 2003 and the Middle East respiratory syndrome virus (MERS-CoV) found in 2012. Whole-genome sequencing and genetic analysis of the strain has demonstrated that the virus is a different branch from SARS-CoV and MERS-CoV [1]. Currently, the epidemic that originated in Wuhan is ongoing. On February 11, 2020, the International Committee on Taxonomy of Virus (ICTV) officially named 2019-nCoV as “severe acute respiratory syndrome coronavirus 2” (SARS-CoV-2) [2]. Furthermore, the World Health Organization (WHO) has named the disease caused by infection with this virus as Coronavirus Disease 2019 (COVID-19). Generally, the incubation period of COVID-19 ranges from 1 to 14 days, with most people developing symptoms between 3–7 days; however, the longest incubation period can reach 24 days [3]. The clinical severity of COVID-19 varies greatly, from asymptomatic to death.

Currently, a positive result in nucleic acid testing (NAT) using reverse-transcriptase polymerase-chain-rection (RT-PCR) technology is the gold standard for diagnosing COVID-19. The assay has high specificity, but low sensitivity. Hence, chest CT has become a critical diagnostic tool for COVID-19, used in close combination with clinical manifestations and epidemiological evidence for disease confirmation. However, discrepancies between the results of NAT and imaging features have been reported in the literature. For example, the CT images of some patients showed apparent lesions in the lungs, yet NAT repeatedly showed negative results until eventually turning positive [4]. Recently, Fang et al. observed that in diagnosing COVID-19, the sensitivity of CT (98%) was significantly higher than that of RT-PCR (71%) [5]. China’s *Diagnosis and Treatment Plan for Novel Coronavirus*-*infected Pneumonia (5th Trial Edition)* has established CT imaging as the clinical diagnosis in Hubei Province. Therefore, there is an urgent need for further studies to provide data and evidence on the manifestations of COVID-19 in chest CT imaging.

In this study, the clinical manifestations, epidemiology, laboratory test results, and chest CT findings of 131 confirmed COVID-19 patients were summarized and investigated, to improve the understanding of the disease, thereby achieving effective control of the epidemic through early diagnosis, prompt treatment, and quarantine, beneficial to the timely implementation of measures for monitoring public health.

## Materials and methods

### General information

Relevant data of COVID-19 patients admitted to 3 hospitals during the period from December 28, 2019 to February 10, 2020 were retrospectively collected. The inclusion criteria were as follows: (1) patient tested positive for NAT using RT-PCR; (2) patient received the first chest CT examination after admission to hospital. A total of 131 patients were finally enrolled in the study (Table 1), including 63 males and 68 females, aged between 20–90 years. The average age of all the patients was 47 ± 15 years, with males averaging at 45 years and females at 49 years. The time interval between disease onset and admission to hospital ranged between 1 and 19 days, with an average of 4.6 days. The results of a series of laboratory tests were recorded, including the white blood cell count (WBC) (normal range 3.5–9.5 × 10^9^/L), neutrophils (normal range 1.8–6.3 × 10^9^/L) and lymphocytes (normal range 1.1–3.2 × 10^9^/L), procalcitonin (normal range 0–0.07 ng/ml), and C-reactive protein (CRP) (normal range 0–10 mg/L).

**Table 1:** Baseline characteristics of the overall study population

Variable	Patients (n = 131)
Age (years)	20-90
Sex	
M	63 (48%)
F	68 (52%)
Exposure history	
Close contact	100 (76%)
Uncertainty	31 (24%)
Clinical symptoms	
Fever	85 (65%)
Cough	85 (65%)
Feeble	13 (10%)
Shortness of breath	5 (4%)
Muscle ache	2 (2%)
Diarrhoea	1 (1%)
Blood biochemistry	
Leucocytes (× 10^9^per L)	
Decreased	11 (8%)
Normal	111 (85%)
Increased	9 (7%)
Neutrophils (× 10^9^ per L)	
Decreased	5 (4%)
Normal	109 (83%)
Increased	17 (13%)
Lymphocytes (× 10^9^ per L)	
Decreased	74 (57%)
Normal	57 (43%)
Procalcitonin (ng/mL)	
Increased	69 (53%)
Normal	62 (47%)
C-reactive protein (mg/L)	
Increased	75 (57%)
Normal	56 (43%)

### Examination methods

All patients were required to wear masks and walk to the CT scan facility using a hallway exclusively for outpatients with fever. Disposable bed sheets were used during each examination. CT scans were performed using the SOMATOM Definition AS + 64 row spiral CT (Siemens, Germany) (First Affiliated Hospital to Army Medical University and Affiliated Hospital of North Sichuan Medical College), and Emotion 16 row spiral CT (Siemens, Germany) (Chongqing Three Gorges Central Hospital) Scan parameter settings were as follows: tube voltage at default machine settings, with automatic tube current, layer thickness ranged at 2–5 mm, and layer spacing ranged at 2–5 mm. The scan covered the area from the thoracic inlet to the costophrenic angle. As patients with relatively severe symptoms found it difficult to hold their breath, the scan was performed in a reverse direction from the costophrenic angle to the thoracic inlet. The lung window was reconstructed with a high-resolution algorithm at 1.0 mm. Sagittal and coronal reconstructions were performed using post-processing workstations. The machine and the room were thoroughly disinfected after the examination of each patient.

### Image analysis

The chest CT of each patient was independently reviewed by two radiologists with 5 and 10 years of experience in CT diagnostics. In case of any discrepancy, a consensus was reached through discussion. The images were analyzed for the following aspects: (1) presence of ground-glass opacities: defined by increase in lung density but without covering the pulmonary blood vessels and bronchial walls; (2) presence of lung consolidation: defined by higher density than ground-glass opacities and blurred margins of pulmonary blood vessels and bronchial tubes; (3) presence of nodular/cord-like shadows; (4) interlobular septal thickening, thickening of vascular, and air bronchogram signs inside the lesions; (5) number of lesions (recorded as 1, 2, 3, and > 3; numbers > 3 were recorded as multiple without the specific number); (6) lesion site (left and right lobe, center of the lung, near the hilum/peripheral: far from the hilum); (7) complications other diseases such as pleural thickening, hydrothorax, pericardial effusion, or lymphadenopathy (short diameter ≥ 1 cm); (8) disease progression: defined by comparing the scope, quantity, and density of lesions detected in two chest CT scans. Obviously fusion of lesions, new lesions, and (or) increased lung density was considered progression. Conversely, obviously reduced lesion size, number, and (or) density was considered absorption. If no significant difference in the lesions between the two CT examinations was observed, the patient’s condition was considered stable.

## Results

### Findings of first chest CT scan

With the 6 cases were negative in the chest CT, 125 of 131 patients presented abnormal. Bilateral involvement was identified in 104 cases (79%), 14 cases (11%) showed lesions in the right lung, while 7 cases (5%) reported lesions in the left lung. 16 cases (12%) involved one lobe, 12 cases (9%) involved two lobe, 13 cases (10%) involved three lobe, 16 cases (12%) involved four lobe, and 68 cases (52%) involved five lobe; The lesions were peripherally distributed in 100 cases (76%), occurred in both peripheral and central locations in 24 cases (18%), and in the center in 1 case (1%). Multiple lesions (> 3) were detected in 115 cases (87%), 1 case (1%) presented 3 lesions, 3 cases (2%) showed 2 lesions, and 6 cases (5%) reported 1 lesion.

**Fig. 1:**
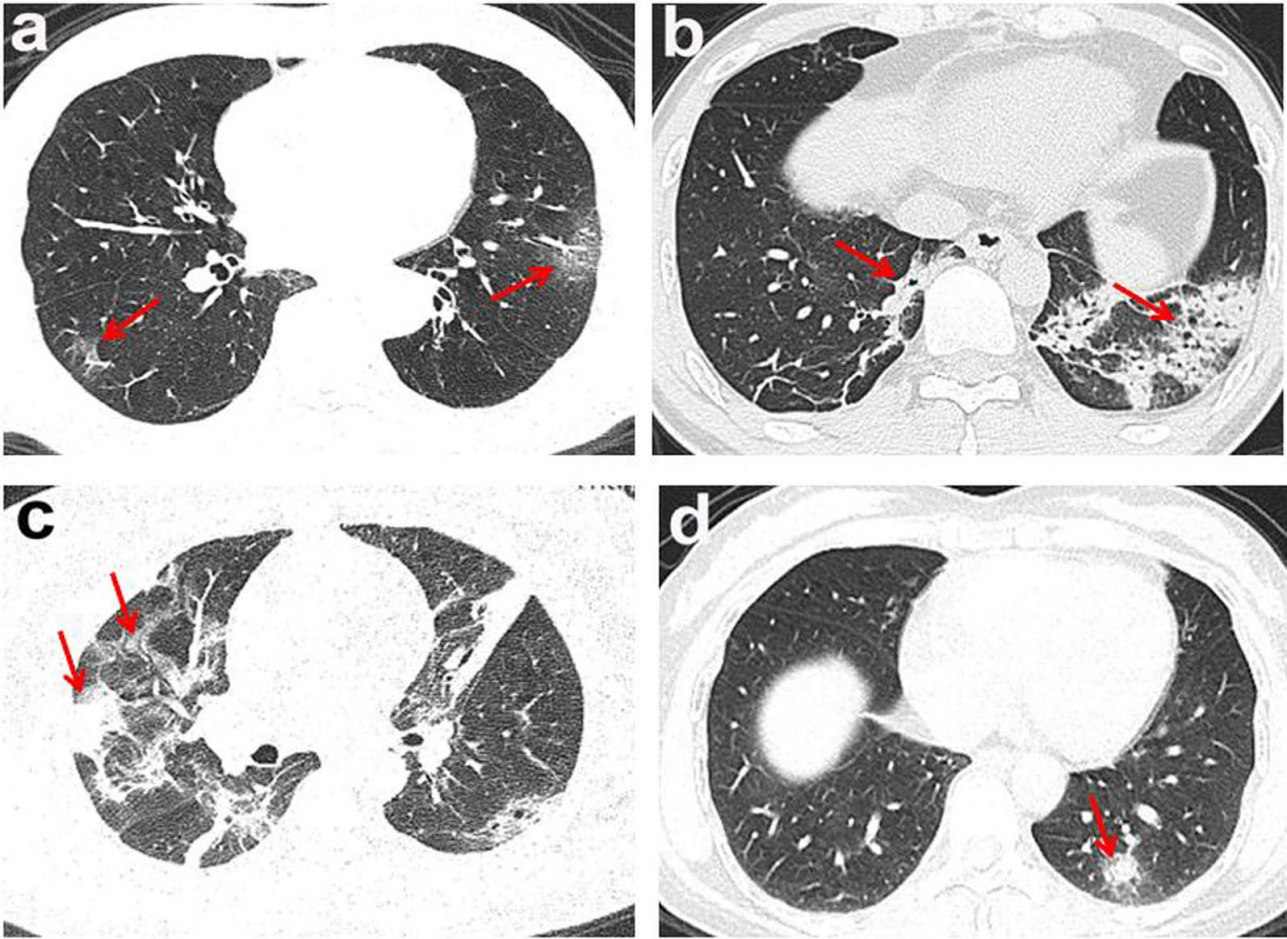
The lesion of multiple morphologic manifestations. The red arrows and boxes indicated the abnormalities. **a** Ground glass opacities; **b** consolidation; **c** consolidation with ground glass opacities; **d** solid nodule (red arrow)

Chest CT imaging manifestations of the 125 positive cases included 106 cases (81%) with patchy ground-glass opacities, 91 cases (69%) with patchy consolidations, 40 cases (31%) with nodules, and 94 cases (72%) with two or more forms of lesions co-existing (Fig. 1a–d). The lesion margins were clear in 60 patients (46%) but blurry in the remaining 65 cases (50%). 68 cases (52%) demonstrated interlobular septal thickening (8 cases presented typical “crazy paving” pattern). Thickening of vascular, air bronchogram signs, or fibrous foci (Table 2). In 1 case, a consolidation with cavity in the right lower lobe. In 3 cases, “reverse halo signs” were detected, and the initial chest CT of 1 case presented nodules, the “reversed halo sign” appeared in the 2 days follow up re-examination in the left lower lobe.

**Table 2:** Innitial chest CT findings of 125 patients

Morphology	Patients (n = 131)
Ground-glass opacities*	20 (15%)
Consolidation*	4 (3%)
Nodule*	7 (5%)
Ground-glass opacities and consolidation	61 (47%)
Ground-glass opacities and nodule	7 (5%)
Consolidation and nodule	8 (6%)
Both of all	18 (14%)
With others	
Interlobular septal thickening	68 (52%)
Vascular enlargement	84 (64%)
Air bronchogram	75 (57%)
Fibrosis	43 (33%)
Pleural thickening	31 (24%)
Hydrothorax	3 (2%)
Lymph node enlargement	17 (13%)

*Without the other two morphology

### Findings of second chest CT scan

**Fig. 2:**
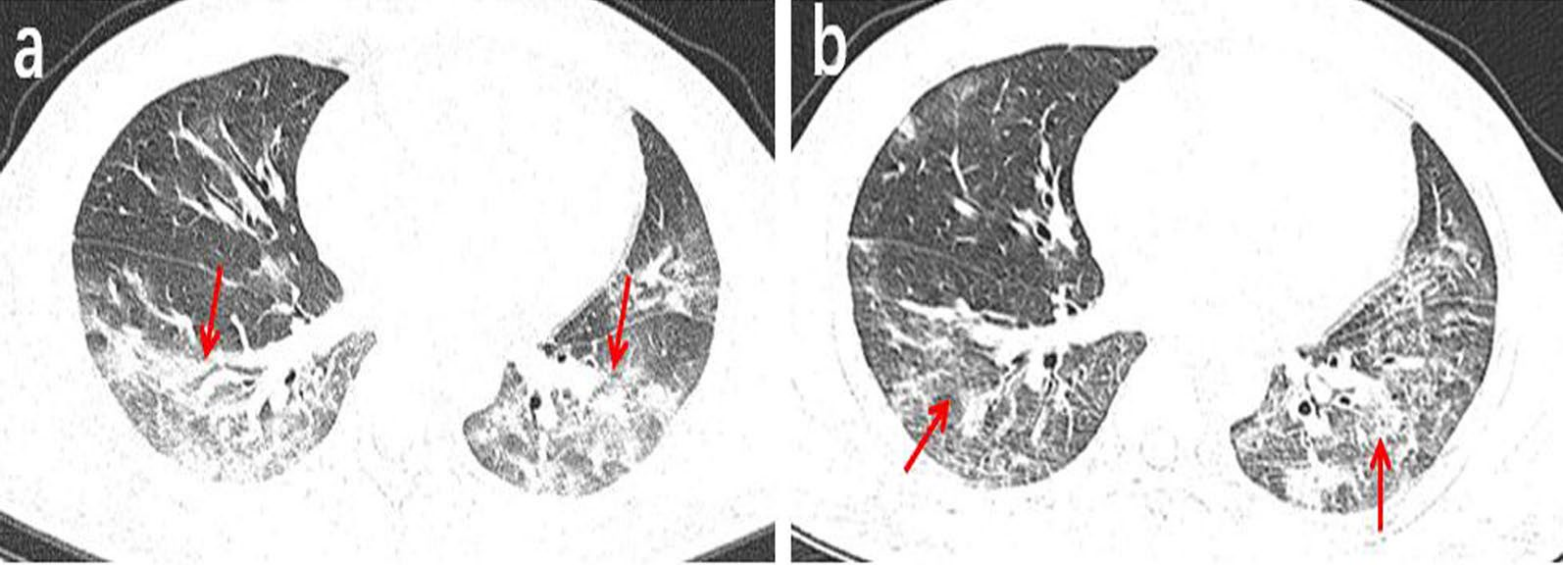
A 38-year-old male working at a hotel presented with a cough, fever, and fatigue for 10 days. **a** The first axial-view chest CT shows diffused, mixed shadows of ground-glass opacities and consolidations (red arrows) with blurred margins. **b** In the second axial-view chest CT scan conducted 2 days after the first one, the lesion density is significantly reduced and the scope of lesions is narrowed (red arrows)

**Fig. 3:**
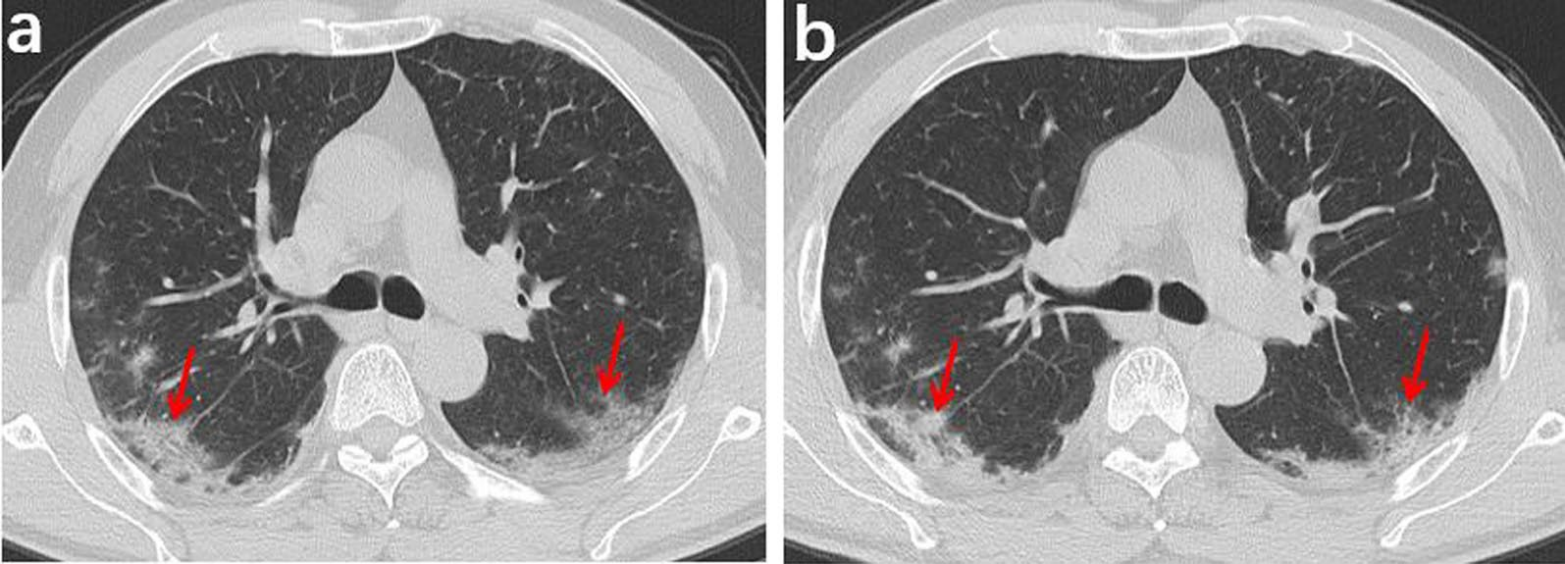
A 55-year-old male who had close contact with people in Wuhan presented fever for 8 day. **a** The initial axial chest CT shows mixed shadows of ground-glass opacities and consolidations with peripherally distributed (red arrows). **b** The follow-up axial-view chest CT shows no significant changes after 4 days later (red arrows)

**Fig. 4:**
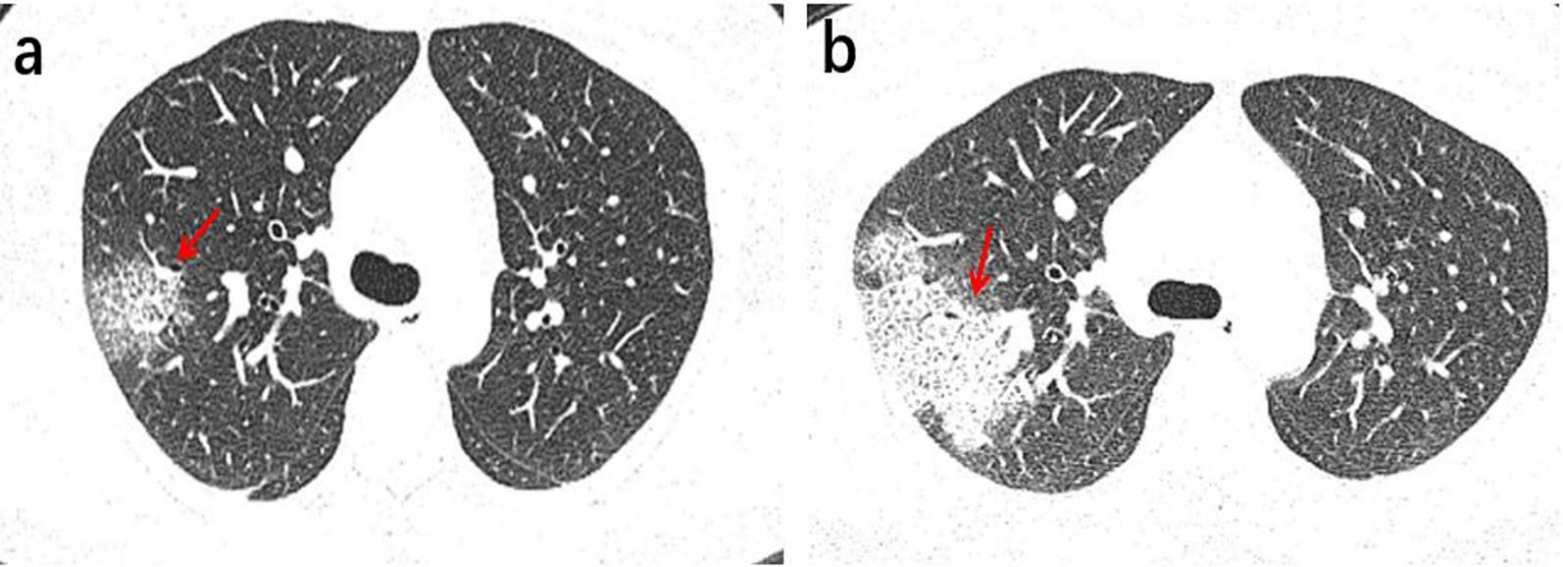
A 46-year-old female who had close contact with people in Wuhan presented fever for 1 day. **a** Axial-view chest CT shows ground-glass opacities in the upper right lobe with clear margins and visible interlobular septal thickening inside, forming the “crazy paving sign” (red arrow). **b** Follow-up axial-view chest CT scan 3 days after the first one shows that the scope of lesions increases significantly, and the lesion density also increases, along with significant thickening of the intralobular and interlobular septa (red arrow)

**Fig. 5:**
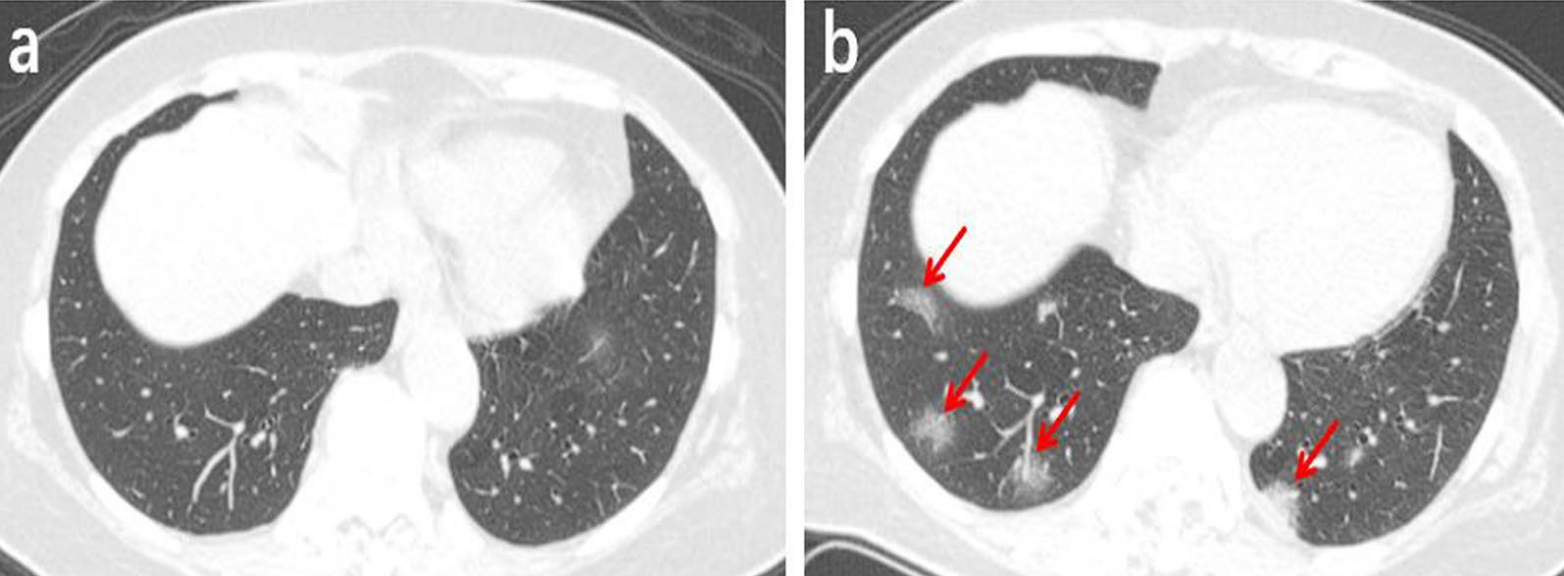
A 64-year-old female Wuhan resident presented with fever and coughs for 3 days. **a** The first axial-view chest CT scan shows no lesions. **b** The second axial-view chest CT scan conducted 4 days after the first CT shows the presence of multiple ground-glass opacities in the lower lobe, located below the pleura with clear margins. Thickened vascular are visible inside the lesions in the lower right lobe (red arrows)

Among the 131 patients, 91 underwent a second chest CT scan (Table 3). The time interval between the two scans ranged from 2 to 7 days, with an average of 3.5 days. In the follow-up, 25 patients (27%) presented absorbed lesions (Fig. 2), The chest CT images of another 25 patients (27%) showed no significant changes (Fig. 3). Disease progression was observed in 41 cases (46%) (Fig. 4), including the 3 patients with negative results in the first scan and new lesions in the second scan performed 2, 4 and 5 days after the first scan (Fig. 5).

**Table 3:** The follow-up results of chest CT in 91 patients

Follow-up results(n)	Innitial chest CT findings	Patients (n = 91)
Absorb (25)	Ground-glass opacities	2 (8%)
	Consolidation	1 (4%)
	Ground-glass opacities and consolidation	17 (68%)
	Ground-glass opacities and nodule	1 (4%)
	Consolidation and nodule	1 (4%)
	Both of all	3 (12%)
Stable (25)	Ground-glass opacities	3 (12%)
	Nodule	2 (8%)
	Ground-glass opacities and consolidation	13 (52%)
	Consolidation and nodule	4 (6%)
	Both of all	3 (12%)
Advance (41)	Ground-glass opacities	9 (22%)
	Consolidation	2 (5%)
	Nodule	4 (10%)
	Ground-glass opacities and consolidation	14 (34%)
	Ground-glass opacities and nodule	5 (2%)
	Both of all	4 (10%)
	(-)*	3 (7%)

*The first chest CT examination was negative

## Discussions

SARS-CoV-2 is a single-stranded RNA virus that can be transmitted from human to human; comparable to the transmission of SARS and MERS, SARS-CoV-2 is also transmitted primarily through respiratory droplets and contact [6, 7]. In this study, we had marginally more female subjects than male subjects, which could be related to the fact that the average age of men was lower than that of women. The major clinical symptoms observed included fever (65%), cough (65%), consistent with previous reports [3]. Notably, 76% of the patients had a history of close contact with people living in Wuhan. Although the contact history of the other 24% was unclear, these patients worked in crowded environments where numerous people gathered (such as restaurants and hotels).

In this group of patients, the WBC and neutrophil counts and the procalcitonin levels were normal in most cases, but the CRP was elevated, consistent with the report by Chen et al. [8]. Lymphocyte count was decreased in 74 patients (57%), and the average age were 51 ± 16 years; 57 patients (43%) presented normal lymphocyte counts, and the average age were 42 ± 11 years(*P *= 0.002). The results indicated that with increased patients’ age the immune cells were more susceptible to damage by the virus, and hence their immunity was weakened [9].

Initial chest CT scan failed to reveal any lesions in 6 of the 131 patients, while the CT findings of the remaining 125 cases showed that the majority of lesions (79%) involved bilateral lungs, 88% of the lesions involved two or more lobes simultaneously, and nearly 76% of the lesions were distributed in the periphery of the lung. The lesions were not confined to certain segments of lung lobes, possibly due to the small size of the virions that tend to deposit on the lobules in the periphery of the lung, thereby causing damage to the alveolar epithelium and affecting multiple adjacent lobules [10].

Common chest CT manifestations of COVID-19 included: (1) patchy ground-glass opacities with clear margins and visible interlobular septal thickening inside the lesions were observed in 106 cases (81%), some cases appeared a typical “ crazy paving pattern”, and visible vascular thickening. This can be explained by the virus-induced diffuse alveolar wall injury, vascular congestion, and alveolar septal inflammation [11]. (2) Increased lesion density, along with disease progression manifested as patchy consolidations, was detected in 91 cases (69%). These pathological changes could be attributed to alveolar wall collapse, causing the replacement of alveoli by exudates or products of other diseases (such as cells and epithelium) [12]. Recently, the autopsy of the first COVID-19 patient who died in China revealed significant shedding of the alveolar epithelium and the formation of the pulmonary hyaline membrane [13], consistent with the clinical symptoms of COVID-19, i.e. cough without substantial sputum. (3) Mixed lesions of ground-glass opacities and consolidations was observed in 79 patients (61%), with or without nodule. Diffused, bilateral pulmonary lesions similar to “white lungs” was observed in 10 patients. In these cases, consolidations constituted most of the lesions, with a small percentage demonstrating complicated pulmonary fibrotic foci, which might be related to the fact that the lesions were in the repairing phase [14].

Uncommon chest CT manifestations of COVID-19 included: (1) nodular lesions in 40 patients (31%). Only 7 of them had simple nodular lesions, whereas the rest had mixed nodular lesions with either ground-glass opacities or consolidations, and the other two signs are the main manifestations. (2) CT showing low central but high peripheral lesion density, or the so-called “reversed halo sign”, in the first CT scan of 1 patient. In another patient, the first CT scan showed nodular lesions, but the “reversed halo sign” emerged in the follow-up CT scan 2 days after. The pathological mechanism remains unknown, but this is not a specific imaging characteristic of COVID-19. Other diseases such as organizing pneumonia, cryptococcosis, and tuberculosis can also present the “reversed halo sign” with different pathological mechanisms [15]. (3) A small number of cases showed thickening of adjacent pleura, hydrothorax, pericardial effusion, and enlarged mediastinal lymph nodes, consistent with previous reports [16]. (4) In 1 case, a consolidation with cavity, it might be related to the bronchial discharge of necrotic material of the lesion. This was an extremely rare phenomenon.

Although the time interval between the two CT examinations for the 91 evaluated cases was short (average 3.5 days), but 66 (73%) patient’s chest CT results demonstrated rapid changes, appear significant progress or absorption. The other 25 (27%) cases was stable. From Table 3, it should note that mixed ground glass and consolidation with or without nodules in the first CT scan. These patients can be progressed, absorbed or stable. However, initial pure patchy ground-glass density imaging indicates a disease aggravated. The risk factors of disease progression are ambiguous. Recently, Liu [17] and Guo [18] have both reported that low lymphocyte counts and hypertension are predictors for worsening conditions, which could be related to the damaged immune system.

The imaging manifestations of COVID-19 are markedly similar to those of SARS and MERS [19, 20]. (1) SARS often presents as single lesion involving unilateral lung, and septal thickening is evident after the 2nd week [21]. (2) MERS is associated with high mortality. It’s difficult to distinguish the imaging from COVID-19, but pleural effusion and pneumothorax are more common in died patients cause by MERS [22]. (3) Early manifestation of influenza virus is primarily tracheobronchitis which presented as nodules or patchy shadows around the bronchial. Centrilobular nodules, pneumatocele formation and lymphadenopathy are often seen in influenza A [23]. To some extent, combined with epidemiology and chest CT can distinguish COVID-19 from other viruses. (4) For mycoplasma pneumonia, the major manifestations include reduced light transmittance of the lung lobes, thickened vascular, thickened bronchial walls, and visible peripheral nodules, presenting the “tree-in-bud” pattern. The disease is commonly complicated by pleural effusion and enlarged mediastinal and hilar lymph nodes [24]. (5) Bacterial pneumonia is often community-acquired or hospital-acquired, with increased WBCs and neutrophils and imaging often showing patchy, nodular, or consolidation shadows distributed along the bronchi or lung segments, which can be distinctly recognized [25]. (6) In cases of cryptococcus infections, lesions are mostly located under the pleura with single or multiple consolidations and nodular shadows, and the disease progresses slowly [26].

The mechanisms during the lesion mainly with ground glass no specific antiviral drugs against progression remain unclear in early stage. We speculate that antiviral drugs may only work at certain times. This phenomenon requires further antivirus study on early intervention. The radiomics will help identify microscopic features of ground glass in early stage. This work may be conducive to differentiate COVID-19 from other viral pneumonia and guide antiviral treatment evaluation.

Our study was limited in the following aspects: (1) Although we summarize the imaging feature of COVID-19, quantitative assessments of different pneumonia lesions need to be done in the future. (2) An in-depth study on whether there is a correlation between the disease course and its imaging manifestations was not conducted due to lack of prognostic data. (3) Not all relevant clinical information was collected. For some patients, the lab tests were incomplete, e.g. liver functions and blood coagulation were not examined.

## Conclusion

In summary, the chest CT manifestations of COVID-19 often presented patchy ground-glass opacities or mixed ground-glass opacities and consolidation, involving the periphery of bilateral lungs, that can quickly change over a short period of time. COVID-19 is highly suspected if the patient also has reduced lymphocytes along with epidemiological evidence. Furthermore, it should be noted that some patients with normal chest CT imaging could demonstrate a positive NAT result.

## Data Availability

All data generated or analyzed during this study are included in this article.

## Abbreviations

COVID-19: Corona Virus Disease 2019
2019-nCov: 2019 Novel coronavirus
SARS: Severe acute respiratory syndrome
MERS: Middle East respiratory syndrome
SARS-CoV-2: Severe acute respiratory syndrome coronavirus 2
WHO: World Health Organization
NAT: Nucleic acid testing
RT-PCR: Reverse-transcriptase polymerase-chain-reaction
